# Supplementary material for: Human Protoparvovirus DNA and IgG in Children and Adults with and without Respiratory or Gastrointestinal Infections
Source: Viruses. 2021 Mar 15;13(3):483. doi: 10.3390/v13030483 (PMC7999311; doi:10.3390/v13030483)

Appendix

Figure S1. qPCR-amplicons of 91 nt from CuV DNA-positive stool and NPA samples.

Alignment of the sequenced 91-nt qPCR amplicons from all the CuV DNA-positive stool and NPA samples in the current study to CuV reference sequence NC_039050.1 (4245-4335 bp within the VP genes). CuV pStBlue was the control plasmid used in qPCR. Y= C or T; M= C or A, K= G or T. Dot (.) indicates identical nucleotides and tilde (~) lacking nucleotides.

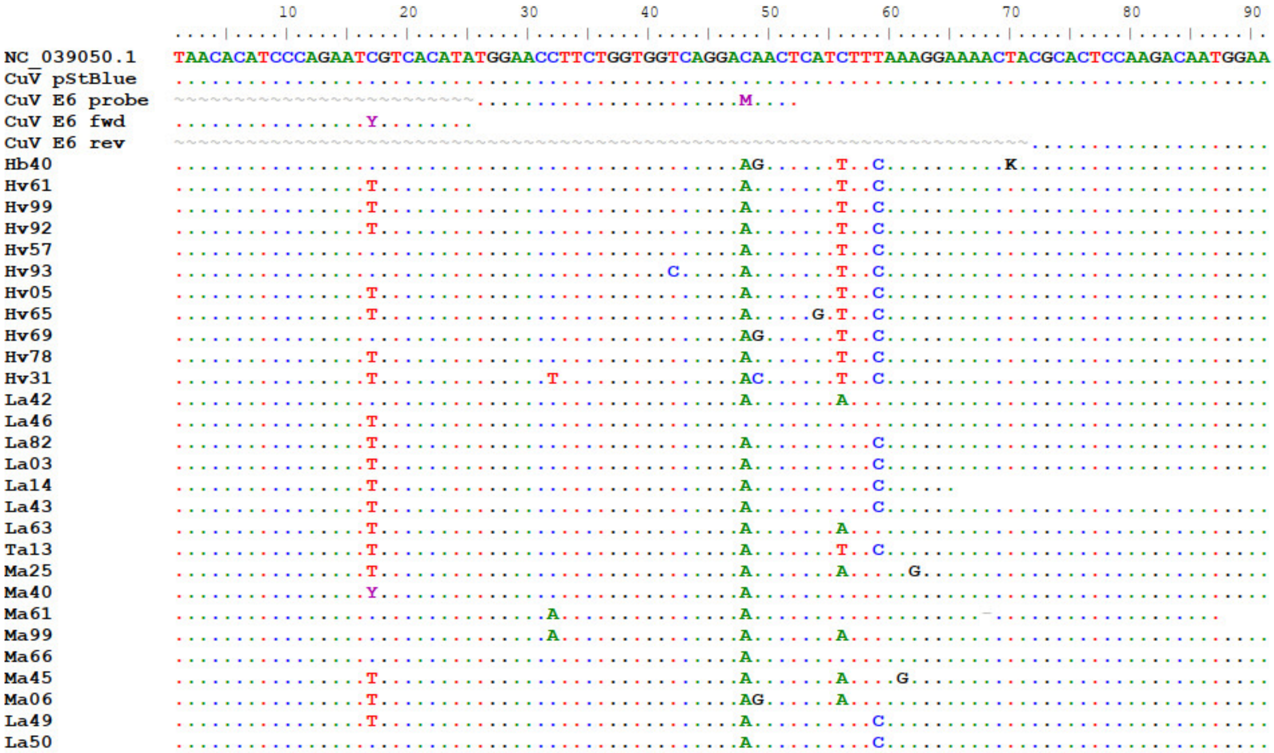

Supplement: Supplementary file 1 [file viruses-13-00483-s001.pdf]
